# Systematic Review of the Role of Biomarkers in Predicting Anastomotic Leakage Following Gastroesophageal Cancer Surgery

**DOI:** 10.3390/jcm8112005

**Published:** 2019-11-17

**Authors:** Cornelius Maarten de Mooij, Martijn Maassen van den Brink, Audrey Merry, Thais Tweed, Jan Stoot

**Affiliations:** 1 Department of Surgery, Zuyderland Medical Center, 6126BG Sittard-Geleen, The Netherlands; martijnmvdb@gmail.com (M.M.v.d.B.); th.tweed@zuyderland.nl (T.T.); j.stoot@zuyderland.nl (J.S.); 2 Department of Epidemiology, Zuyderland Medical Center, 6126BG Sittard-Geleen, The Netherlands; a.merry@zuyderland.nl

**Keywords:** anastomotic leakage, upper gastrointestinal tract, biomarkers

## Abstract

Anastomotic leakage (AL) following gastroesophageal cancer surgery remains a serious postoperative complication. This systematic review aims to provide an overview of investigated biomarkers for the early detection of AL following esophagectomy, esophagogastrectomy and gastrectomy. All published studies evaluating the diagnostic accuracy of biomarkers predicting AL following gastroesophageal resection for cancer were included. The Embase, Medline, Cochrane Library, PubMed and Web of Science databases were searched. Risk of bias and applicability were assessed using the Quality Assessment of Diagnostic Accuracy Studies (QUADAS) 2 tool. Twenty-four studies evaluated biomarkers in the context of AL following gastroesophageal cancer surgery. Biomarkers were derived from the systemic circulation, mediastinal and peritoneal drains, urine and mediastinal microdialysis. The most commonly evaluated serum biomarkers were C-reactive protein and leucocytes. Both proved to be useful markers for excluding AL owing to its high specificity and negative predictive values. Amylase was the most commonly evaluated peritoneal drain biomarker and significantly elevated levels can predict AL in the early postoperative period. The associated area under the receiver operating characteristic (AUROC) curve values ranged from 0.482 to 0.994. Current biomarkers are poor predictors of AL after gastroesophageal cancer surgery owing to insufficient sensitivity and positive predictive value. Further research is needed to identify better diagnostic tools to predict AL.

## 1. Introduction

Surgical resection is considered the mainstay of treatment for the management of advanced cancer of the esophagus, gastroesophageal junction and stomach [1,2]. Anastomotic leakage (AL) following these resections is a feared life-threatening complication associated with increased morbidity, mortality, prolonged hospital stay and considerably increased health care costs [3,4,5,6,7,8]. Despite continual advancements in the multimodal treatment of these cancers, AL remains a common postoperative complication with incidences ranging from 0 to 40 per cent [8,9,10,11,12,13]. An important factor explaining the wide range of the incidence of AL is the large variety in the definition of this complication throughout the literature [14]. Although the exact etiopathogenesis of AL has not yet been clarified, numerous risk factors for developing this complication have been recognized [15,16,17]. Among others, preoperative weight loss, perioperative blood loss, and longer operative time have been shown to be persistent risk factors across different studies [16,18,19,20]. It remains, however, difficult to individually predict AL in each patient.

With the implementation of enhanced recovery after surgery (ERAS) protocols for patients who undergo gastroesophageal cancer surgery, oral nutrition is offered as soon as postoperative day (POD) 1 and patients are discharged on average between POD 6 and 12 [21,22,23]. AL can occur after discharging the patient and this increases the danger of a late diagnosis [24]. Therefore, early suspicion of AL is paramount to exclude patients from enhanced recovery pathways with early postoperative oral feeding, since this can prevent further aggravation into a fulminant sepsis, multiple organ failure or death [25]. Moreover, a timely diagnosis of AL can optimize its treatment and may have a beneficial effect on quality of life, disease-free survival and overall survival [26].

A biomarker is defined as a naturally occurring characteristic that is measured objectively as an indicator of normal biological processes, pathogenic processes, or pharmacologic responses to a therapeutic intervention [27]. Multiple biomarkers indicating various stages of ischemia, inflammation, and necrosis have been investigated extensively in their ability to predict or diagnose AL following gastroesophageal cancer surgery. Since the first results on this subject were published in 1996 on predicting and diagnosing AL through levels of amylase in drain fluids, research has extended widely and lately there has been more attention for acute phase proteins like C-reactive protein (CRP) or markers of intestinal cell damage such as procalcitonin (PCT). To the best of our knowledge, this is the first systematic review that aims to assess biomarkers for their use in the (pre)clinical detection of AL in the early phase after gastroesophageal cancer surgery.

## 2. Experimental Section

### 2.1. Literature Search

To identify all primary diagnostic test accuracy studies that evaluated the role of biomarkers in predicting AL after esophagectomy, esophagogastrectomy or gastrectomy, an extensive review of the literature was conducted according to the guidelines in the Preferred Reporting Items for Systematic Reviews and Meta-Analysis (PRISMA) statement [28]. The MEDLINE, Embase, PubMed and Web of Science databases were searched independently by two authors from January 1990 to July 2019. The last search was run on 6 July 2019. The search terms were: anastomotic leak* OR anastomotic complication* OR anastomotic dehiscence AND biomarker* OR marker* OR drain OR serum OR plasma AND gastrectom* OR esophagectom* OR esophagogastrectom* OR upper gastrointestinal*. Reference lists of all relevant papers were searched manually to identify further relevant studies. Only studies in humans and articles written in English were included. Other (systematic) reviews were excluded.

### 2.2. Study Selection

All studies evaluating biomarkers in the context of AL following resection for cancer of the esophagus, esophagogastric junction, or stomach were included in this review. No restriction concerning the type of biomarker was imposed. All studies, with a clear index test, reference standard, sensitivity and specificity were included in this systematic review. Eligible studies were assessed independently by two authors (C.M.d.M. and M.M.v.d.B.) and any disagreement over in- or exclusion was resolved by the intervention of a third author (T.T.).

### 2.3. Data Extraction

Data extraction was performed independently by two authors and entered into predefined tables—disagreements were resolved in a consensus conference. The main outcome of interest was AL, as defined in the included studies. The first author’s name, study design and period, type of approach, AL rate, neo-adjuvant chemoradiation therapy, diagnostic accuracy of the studied biomarker(s), used cut-off value(s) and POD of measurement were recorded. The diagnostic accuracies of the different biomarkers were described with sensitivity, specificity, positive predictive value, negative predictive value, likelihood ratios and/or area under the receiver operating characteristic (AUROC) curve [29].

### 2.4. Quality Assessment

For all included studies, their quality was assessed independently by two authors (C.M.d.M. and M.M.v.d.B.) using the Quality Assessment of Diagnostic Accuracy Studies (QUADAS) 2 tool [30]. This validated tool assessed risk of bias and concerns about applicability by evaluating four key domains: patient selection, index test, reference standard, and flow of patients through the study and timing of tests.

### 2.5. Statistical Analysis

Due to the high heterogeneity between and within the studies, no meta-analysis was performed. Heterogeneity was caused by large differences regarding the cut-off values and time of the postoperative measurements both between and within the studies. As a result, no paired forest plots or summary receiver operating curves (sROCs) were computed [31]. Instead, descriptive tables were used to provide a clear overview of the diagnostic accuracies of the primary studies. An epidemiologist was consulted before omitting the meta-analysis.

## 3. Results

### 3.1. Study Selection

**Figure 1:**
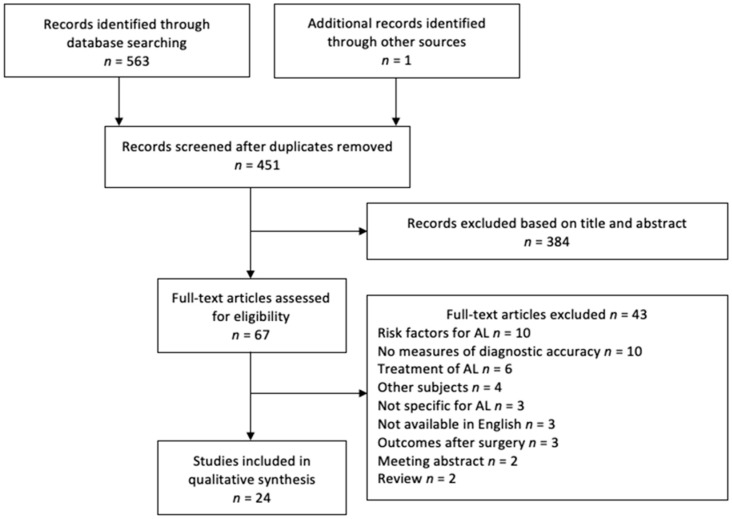
PRISMA (Preferred Reporting Items for Systematic Reviews and Meta-Analysis) flow diagram showing selection of articles for review. AL, anastomotic leakage.

A PRISMA flowchart showing the selection of articles for this systematic review is presented in Figure 1. In total, 24 studies met the inclusion criteria.

### 3.2. Study Characteristics

Study characteristics of the included studies are shown in Table 1. The role of systemic biomarkers was evaluated separately in fifteen studies, [32,33,34,35,36,37,38,39,40,41,42,43,44,45,46] drain fluid in nine [33,44,45,46,47,48,49,50,51], combined scoring systems in three studies [35,41,52], and mediastinal microdialysis [53], urine volatile organic compounds [54] and respiratory index [40] were all evaluated in one study. All studies except three [37,53,54] were retrospective. All studies included operations for upper gastrointestinal cancer. Only ten studies reported on the rate of elective surgery, in which it was mostly 100 per cent elective [32,35,36,37,43,47,48,53,54] except for Dutta and colleagues in which surgeries were 97% elective [34]. While some studies completely or partially operated through a minimally invasive approach, the majority of the resections were performed through an open approach. Different methods and materials were used to measure all biomarkers.

### 3.3. Validity Assessment

**Figure 2:**
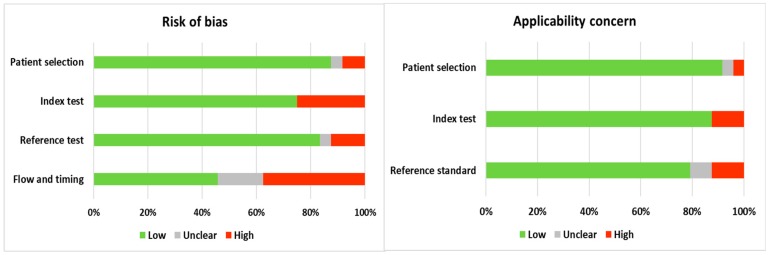
Quality assessment of Diagnostic Accuracy Studies 2 (QUADAS-2). Proportion of studies with low, high, or unclear risk of bias.

Figure 2 shows the results from the QUADAS-2 assessment [30]. Overall, the risk of bias is low, and the applicability is good. However, there may be bias due to the open approach used in the majority of patients. Furthermore, the lack of a standard reference test and the time interval between the index test and the reference standard is not optimal. Only one study reported blinding [37].

### 3.4. Definition of Anastomotic Leakage

Various definitions of AL were found in the included studies, as shown in Table 2. Six studies [40,42,43,45,49,50] did not mention the median or range concerning the day of AL diagnosis. In the remaining eighteen studies AL was diagnosed with a median on POD 7, ranging between 1 and 30 days. The AL rate ranged from 6.7 per cent [55] to 66.2 per cent [40].

### 3.5. Diagnostic Accuracy Biomarkers

In total, twenty-four different biomarkers were investigated for their diagnostic accuracy in detecting AL. The most commonly evaluated biomarkers were C-reactive protein, leucocytes and amylase. Biomarkers were divided into four main categories; (1) systemic biomarkers of inflammation, (2) ischemia, (3) peritoneal fluid biomarkers and (4) combined scores of biomarkers. Table 3 shows a summary of CRP measurements with very good diagnostic accuracy with an AUROC higher than 0.800. Table 4 shows a summary of all other biomarkers (except CRP) with good diagnostic accuracy with an AUROC higher than 0.700. All postoperative levels of biomarkers were raised albeit at different time points and with significant variation. Biomarkers without any significant diagnostic accuracy are not shown in Table 3 and Table 4. Reported ranges of test characteristics were derived from those studies, which reported statistically significant diagnostic accuracy of included biomarkers.

#### 3.5.1. Biomarkers of Inflammation

Thirteen studies [32,33,34,35,36,37,39,40,41,42,43,44,45] evaluated fourteen different inflammatory biomarkers for detecting AL, namely CRP, leucocytes, procalcitonin (PCT), albumin, prealbumin, per centage of neutrophils, fibrinogen levels, urinary volatile organic compounds (VOCs), interleukin (IL) 2R, IL-6, IL-8, IL-10, tumor necrosis factor (TNF) α and blood G antigenemia (BG).

CRP was evaluated in twelve studies [32,34,35,36,37,39,40,41,42,44,45,50], with a total of 1818 patients—of whom, 243 developed AL. In eight of these studies [32,34,36,37,39,41,42,44] CRP was found significant in predicting AL. CRP levels were measured between POD 1–7, mostly on POD 3, and were typically significantly elevated several days before the diagnosis of AL. The reported cut-off values of CRP ranged from 78 to 229 mg/L between POD 1 and 5. Sensitivity ranged from 55 to 100 and specificity from 42 to 100 per cent with AUROCs from 0.648 to 0.994. The highest diagnostic accuracy was established by Ji and colleagues [39], who reached an excellent AUROC of 0.994 at a cut-off value of 117 mg/L on POD 1, yielding a sensitivity of 90.0 and a specificity of 89.0 per cent.

Leucocytes were evaluated in seven studies [32,33,35,37,40,41,42], with a total of 943 patients—of whom, 169 developed AL. In four of these studies [32,33,35,41], leucocytes were found statistically significant in predicting AL. Leucocytes levels were measured on POD 1–10, but mainly on POD 3–5. The reported cut-off values ranged from 6.89 × 10^9^/L to 15.0 × 10^9^/L, resulting in a sensitivity ranging from 6.0 to 94.0 per cent and a specificity from 21.0 to 97.0 per cent. No study reported good diagnostic accuracy for leukocytes. Reported AUROCs were found between 0.625 and 0.715, which can be considered as sufficient to good. Noble and colleagues [41] reported the highest AUROC of 0.715 using a cut-off value of 8.95 × 10^9^/L on POD 5, resulting in a sensitivity of 78.0 and specificity of 58.0 per cent.

PCT was evaluated in three studies [32,37,40], with a total of 359 patients—of whom, 86 developed AL. PCT levels were measured on POD 1, 3, 5, and upon admission at the intensive care unit (ICU). The reported cut-off values ranged from 0.38 to 3 ng/mL and resulted in a sensitivity ranging from 22.0 to 78.0 and a specificity ranging from 62.0 to 100.0 per cent. The negative predictive values (NPVs) ranged from 64.0 to 94.0 per cent and positive predictive values (PPVs) from 19.0 to 100 per cent. Diagnostic accuracy, described with the AUROC, ranged from sufficient at 0.672 to very good at 0.860. Hoeboer and colleagues [37] reported the highest diagnostic accuracy with an AUROC of 0.860 and a cut-off value of 0.86 ng/mL on POD 3, giving a sensitivity of 67.0, specificity of 80.0, NPV of 87.0, and a PPV of 55 per cent.

Albumin was evaluated in three studies [35,40,41], with a total of 416 patients—of whom, 87 developed AL. In two of the three studies [40,41], albumin was found statistically significant in predicting AL. Albumin levels were measured on POD 1–7 and on admission at the ICU. The reported cut-off values ranged from 23 to 31 g/L, resulting in ranges of sensitivity from 34 to 76 and specificity from 56 to 92 per cent. The only AUROC reported by Noble and colleagues was 0.742 at a cut-off value of 22.5 g/L, with a sensitivity of 76 and a specificity of 56 per cent. This can be considered as good diagnostic accuracy.

Prealbumin was evaluated by Goa and colleagues [45], with a total of 96 patients—of whom, 12 developed AL. Prealbumin was tested on POD 5 with a cut-off value of 128 g/L, reaching a sensitivity of 100 per cent, specificity of 50 per cent and a very good AUROC of 0.825.

Percentage of neutrophils (PN) was evaluated by Asti and colleagues [32], with a total of 243 patients—of whom, 29 developed AL. PN was measured on POD 3, 5, and 7. On POD 3 and 5 cut-off values of respectively 78.9 and 73.4 per cent were found to be significant in predicting AL. On POD 3 the diagnostic accuracy was sufficient with an AUROC of 0.683 using a cut-off value of 78.9 per cent. This elicited a sensitivity of 77 per cent, a specificity of 57 per cent, positive likelihood ratio (LR+) of 1.77 and a negative likelihood ratio (LR-) of 0.41. The AUROC increased to 0.692 using a different cut-off value of 73.4 per cent on POD 5. This resulted in a sensitivity of 68 per cent and specificity of 71 per cent.

Fibrinogen levels were evaluated by Edagawa and colleagues [44] in their study with a total population of 204 patients—of whom, 44 developed AL. Fibrinogen levels were tested on POD 4 with a cut off value of 712 mg/dl in a test group with a sensitivity and specificity of 52 and 90 per cent, respectively. However, validation of their results in a separate group failed with sensitivity of 17 per cent and specificity of 92 per cent.

Cytokines were evaluated by Song and colleagues [43], with a total of 183 patients—of whom, 16 developed AL. Cytokine levels of interleukin (IL)-2R, -6, -8, -10 and TNF-α were measured on POD 1. The reported cut-off values ranged from 17.2 to 785.4 pg/mL, resulting in a sensitivity ranging from 53 to 100 and specificity from 46 to 85 per cent. Overall, the AUROC of cytokines ranged from sufficient (0.683) to good diagnostic accuracy (0.784), of TNF-α and IL-10 respectively.

Blood G antigenemia (BG) was evaluated by Li and colleagues [40], with a total of 71 patients with acute respiratory distress syndrome admitted to the ICU—of whom, 47 developed AL. BG levels were measured upon admission at the ICU. The reported cut-off value of 93 pg/mL resulted in a sensitivity of 72 per cent, specificity of 83 per cent, an NPV of 72 per cent and a PPV of 67 per cent.

Urinary volatile organic compounds (VOCs) were evaluated by Plat and colleagues [54]. Urinary VOCs reflect the metabolic status of an individual which is associated with a systemic immunological response. Nine patients developed AL in the small study group of 31 patients. The obtained results were not very promising for the detection of AL after esophagectomy, reaching a sensitivity of 54 per cent, specificity of 55 per cent and an AUROC of 0.51 with a *p*-value of 0.88.

In summary, regarding biomarkers of inflammation CRP, PCT, and prealbumin especially show good to excellent diagnostic accuracy in detecting AL in the early phase after gastroesophageal cancer surgery.

#### 3.5.2. Biomarkers of Ischemia

Only three articles [38,40,53] evaluated biomarkers of ischemia as a biomarker for AL. Lactate, pyruvate, glucose, lactate/pyruvate (L/P) ratio, lactate/glucose (L/G) ratio, glucose and blood gas components are possible biomarkers of ischemia. None of the articles reported the AUROCs for these biomarkers.

Ellebaek and colleagues [53] reported a statistically significant increase in the L/P and L/G ratios and multiple cut-off values were computed for early as well as any AL in 54 patients—of whom, seven developed AL. For early AL, which was defined as AL diagnosed between POD 1–4, cut-off values of 105 and 7.9 for respectively L/P ratio and L/G ratio were computed. L/P ratio elicited a sensitivity of 100, specificity of 94, NPV of 100 and PPV of 50 per cent. The L/G ratio resulted in similar diagnostic accuracy with a sensitivity of 100, specificity of 92, NPV of 100, and PPV of 43 per cent. The diagnostic accuracy of these aforementioned biomarkers for predicting any AL were generally worse with a sensitivity of 57, specificity ranging from 79 to 94, NPV from 93 to 94, and PPV of 29 to 57 per cent.

Lactate was evaluated by Ip and colleagues [38] in a total of 136 patients—of whom, 18 developed AL. Lactate levels were measured on POD 1, 2 and 3 with cut-off values of 2.4, 1.7 and 1.0 mmol/L respectively. The highest diagnostic accuracy was reached on POD 2, with a sensitivity of 72 and specificity of 88 per cent.

In summary, regarding biomarkers of ischemia especially those measured with the uncommon procedure of mediastinal microdialysis achieve high sensitivity and specificity. More easily determined serum lactate achieves high specificity on POD 2.

#### 3.5.3. Peritoneal Drain Fluid Biomarker

Amylase derived from peritoneal drain fluid was evaluated in nine studies [33,45,46,47,48,49,50,51,55], with a total of 654 patients—of whom, 73 developed AL. Levels of amylase were measured on POD 1–10. The reported cut-off values of 23 to 1900 IU/L yielded a sensitivity ranging from 21 to 100 per cent and specificity from 48 to 100 per cent. Not all studies reported NPVs, PPVs and AUROCs for all their cut-off values. The highest diagnostic accuracy was reported by Giulini and colleagues [46] and was considered as very good with an AUROC of 0.814. The reported cut-off value of 335 IU/L on POD 1 resulted in a sensitivity of 75 per cent and a specificity of 100 per cent.

In summary, amylase derived from peritoneal drain fluid has good diagnostic accuracy with especially high NPVs.

#### 3.5.4. Combined Scores

Combined scores of inflammatory and ischemic biomarkers were constructed to reach higher diagnostic accuracy and were evaluated in four studies [35,40,41,52]—all of which assessed combinations of at least two biomarkers. Three articles evaluated the Noble and Underwood (NUn) score, a logistic regression model using the inflammatory biomarkers CRP, leucocytes and albumin. The NUn score was evaluated in a total of 561 patients—of whom, 68 developed AL. Measurements were taken between POD 1 and 7, mainly on POD 4. Cut-off values ranged from 7.66 to 10.00, resulting in ranges of sensitivity from 0 to 95 and specificity of 5 to 100 per cent.

AUROCs ranged from sufficient to very good accuracy. Noble and colleagues [41] reported the highest diagnostic accuracy with an AUROC of 0.801, a sensitivity of 95 and specificity of 49 per cent at a cut-off value of 10.

Li and colleagues [40] evaluated the inflammatory biomarker PCT combined with BG. The measurements were taken upon admission to the ICU. The reported cut-off value of 261 elicited a sensitivity of 72, specificity of 92, NPV of 72, and PPV of 92 per cent. The diagnostic accuracy can be considered as very good with an AUROC of 0.870.

In summary, combining biomarkers in a predictive model can have a synergistic effect and can achieve good diagnostic accuracy as shown by different primary studies.

## 4. Discussion

The purpose of this review was to provide an overview of the diagnostic accuracy of biomarkers in predicting AL following esophagectomy, esophagogastrectomy, or gastrectomy for cancer. This systematic review has identified systemic biomarkers as well as biomarkers derived from peritoneal drain fluid and mediastinal microdialysis that were significantly elevated in the presence of AL, albeit at different time points and with different cut-off values. While the biomarkers generally showed poor diagnostic accuracy in predicting AL when assessed individually, combined scores of biomarkers showed improved accuracy.

Despite extensive research in animal models and human studies, the exact pathophysiology of AL remains largely unknown [56,57]. The current hypothesis involves ischemia, inflammation and dysbiosis. In addition, technical aspects of the surgical procedure should also be taken into account [58,59]. Different models, such as the two-wound model or two-hit hypothesis of sepsis fail to completely explain the etiopathogenesis of AL [60]. This gap in knowledge hampers the finding of new leads for biomarkers or treatments [61]. AL can develop early or late in the postoperative period and it is believed that the two occur via different pathophysiologic processes [62]. An early leak is more likely to be the consequence of a technical defect while a late leak could either be an early clinically occult leak or could be the consequence of an increased oral intake upon discharge [33,63]. Regardless of the timing or pathophysiology, both early and late leaks need to be diagnosed or predicted as soon as possible, preferably with a minimally invasive objective tool.

The role of the microbiome is not yet fully elucidated but is suspected to play an important role in the emergence of AL [64]. Moreover, biomarkers such as estimated glomerular filtration rate (eGFR), hemoglobin (Hb) A1c, presepsin and intestinal fatty-acid binding protein (I-FABP), which have already been identified as risk factors for AL or as biomarkers in colorectal AL, should be investigated for their predictive qualities in gastroesophageal AL [65,66,67,68].

CRP and leucocytes are acute phase proteins which are elevated in case of an inflammatory response through infectious and non-infectious causes [69]. Especially on POD 3 and 4 when the inflammatory response of the resection has been attenuated in patients with no complications, an elevated CRP can indicate the presence of a postoperative infectious complication [70,71,72,73]. However, CRP cannot reliably discriminate between surgical and infectious complications, since it is elevated in both circumstances [74,75,76]. Instead, its strength lies in excluding AL on POD 3–5, as it has a useful negative predictive value and can prevent the use of possible harmful swallow studies [32,36]. Similar to CRP, the accuracy of leucocytes as a biomarker for AL lies in excluding rather than indicating this postoperative complication [36,37,74]. Similar conclusions were drawn by a recently published systematic review and meta-analysis by Aolfi and colleagues, who also concluded that CRP may be a useful marker to rule out leakage with reassuring clinical and radiological signs [77].

In contrast to CRP and leucocytes, PCT is believed to be a more specific marker of severe infections and complications [78,79,80,81,82]. Elevated levels of PCT could specifically indicate the presence of combined surgical and infectious complications—of which, AL is the most common [38]. However, overall results have been inconclusive and the discriminatory ability of PCT for different subtypes of postoperative complications remains unknown [79,80,83]. Moreover, PCT is not routinely included in laboratory tests and is more expensive than CRP or leukocytes [37,42].

Ellebaek and colleagues also focused on the ischemic conditions in which AL can occur [53]. Through mediastinal microdialysis, which is a minimally invasive diagnostic technique that is used for continuous measurement of analyte concentrations by measuring the diffusion of compounds over a semi-permeable membrane in the mediastinum. Especially by focusing on markers of ischemia, very high diagnostic accuracy was reached. However, the study population was small, and the measured biomarkers are involved in immune responses of many other inflammatory diseases. Moreover, placing the drain required for the dialysis resulted in a serious adverse event in one patient who required surgical reintervention.

Lactate is elevated in the presence of ischemia and it is one of the contributing factors of AL, Ip and colleagues reported that it has good diagnostic accuracy for detecting this complication [38,84]. However, lactate does not account for AL caused by technical failures. Moreover, hypovolemia can influence the serum levels of lactate.

The NUn score was evaluated in three different studies without establishing a significant external validation [35,41,52]. While a combining score can lead to higher diagnostic accuracy, the results of these scores are not readily available for the physician during clinical activities in contrast to individual biomarkers.

Lastly, amylase is a simple and inexpensive biomarker that showed significant elevation in multiple primary studies [33,47,48,49,50]. However, while amylase could be significantly elevated 2 days prior to conventional AL diagnostic methods, Schots and colleagues [50] stated that this increase is significant from POD 4 onwards. Moreover, amylase is an isoenzyme, which is also used to distinguish anastomotic leak from pancreatic fistula by measuring the salivary-type and pancreatic-type plasma amylase levels.

Current postoperative management is targeted on early discharge and a statistical predictive finding from POD 4 onwards could be too late to predict anastomotic leak before discharge [23]. Moreover, placement of the drain is of great importance when using amylase as a biomarker, since the distance of the drain to the anastomosis influences amylase measurements [47]. Most studies did not report an evaluation of this distance in relation to amylase levels and did not report how or where the drains were placed. Lastly, a recent Cochrane review showed no reductions of postoperative complications with prophylactic drain placement. On the contrary, drains are associated with pain and discomfort around the drain site, increased risk of infection and more analgesic use [85,86]. In line with the recently published Cochrane review and our own experience in accordance with the ERAS guidelines, the use (duration and number) of drains following gastrectomy should be avoided [23]. Regarding esophagectomy, however, the ability of amylase to detect AL in the early postoperative phase could outweigh the morbidity associated with drain use and more research to elucidate the best approach is needed [87].

Recently several publications have focused on the identification of patients who are more likely to develop AL by identifying perioperative risk factors such as diabetes mellitus, preoperative leukocyte count, pre-existent coeliac axis stenosis or perioperative transfusion [88,89,90]. High-risk patients who have one or more of these risk factors should be identified preoperatively and, in these patients, it is useful and justifiable to measure biomarkers more regularly than in low-risk patients. While the sensitivity and specificity of the biomarkers does not differ between low- and high-risk patients, the negative and positive predictive values are affected by the prevalence and a positive test in a high-risk patient is more likely to be an indicator of AL [91]. In these high-risk patients, biomarkers can therefore help in the consideration to use further imaging or even resubmit patients to the operation room.

This review has several limitations. There are small differences between the definitions of AL used in the primary studies, varying from clinical symptoms to any sign of leakage on imaging, endoscopy or reoperation [14]. Moreover, there were inconsistencies in the application of the AL definition, since some studies used imaging routinely while others performed additional research after the emergence of clinical signs. These were sometimes based on the index test such as CRP or drain amylase. The composition of drainage fluid depends on drain location and the use of peritoneal drains has long been debated [85]. The systemic inflammatory response caused by surgery is reduced when using a minimally invasive technique and most patients included in this review underwent open surgery [92,93,94]. In addition, the timing of the measurements varied greatly as some studies used a single measurement while others measured biomarkers on a daily basis. Few of the included studies included a baseline measurement. Considering the location of the anastomosis; intracervical anastomoses are more prone to AL than intrathoracic anastomoses. However, AL from the latter is considered to be more life threatening [95,96,97,98]. The majority of included studies did not report on the location of the anastomotic leak. Lastly, the use of medication can influence the inflammatory response and thus the levels of certain biomarkers. None of the studies took this aspect into account [99].

The findings summarized in this systematic review clearly show that no single biomarker can detect or predict anastomotic leakage with absolute certainty. The strength of the biomarker with the highest diagnostic accuracy, CRP, lies in excluding AL, since it cannot reliably discriminate between surgical and infectious complications. However, individual biomarkers have shown promising results and a synergistic effect established by combining different biomarkers with good diagnostic accuracy such as CRP, PCT, and amylase should be used to predict or detect AL until a new more accurate biomarker has been found.

## 5. Conclusions

Several different biomarkers are involved in the early detection of AL after gastroesophageal surgery for cancer. In general, these biomarkers are poor predictors of AL owing to inadequate sensitivity and positive predictive value. Different diagnostic accuracies were found at a wide range of cut-off values and PODs. Combined scores of biomarkers can lead to higher diagnostic accuracy in the early detection of AL. However, validation studies failed to repeat the significance found in the primary studies. Current biomarkers are useful to distinguish between low-risk patients and patients at high risk for AL, who may have an advantage of further imaging. High quality prospective studies with clear definitions of AL are needed to identify a minimally invasive objective tool, such as a biomarker, that reflects the perianastomotic environment for predicting AL in the early postoperative period before discharge.

## Figures and Tables

**Table 1:** Summary of included studies evaluating biomarkers for detecting anastomotic leakage in gastroesophageal anastomosis.

Reference	Resection	Study Design ^b^	Study Period	MI Approach (%)	Patients (n)	AL (n)	N CRT (n)	Marker Type	Biomarkers
Asti et al. [32]	Esophageal	Retrospective	2012–2017	100	243	29	96	Serum	CRP, PCT, leucocytes and PN
Baker et al. [33]	Esophageal	Retrospective	2009–2014	93	100	13	68	Serum and drain	Amylase and leucocytes
Berkelmans et al. [47]	Esophageal	Retrospective	2013–2014	100	89	15	84	Drain	Amylase
Dutta et al. [34]	Esophagogastric	Retrospective	2005–2009	NR	136	17	80	Serum	CRP
Edagawa et al. [44]	Esophageal	Retrospective	2007–2009	66	204	44	NR	Serum	CRP, FIB
Ellebaek et al. [53]	Esophagogastric	Prospective	2006–2010	0	54	7	29	Mediastinal microdialysis	Lactate, pyruvate, glucose, glycerol ^a^ and pyrovate
Findlay et al. [35]	Esophagogastric	Retrospective	2008–2013	0	248	21	189	Serum and drain	CRP ^a^, leucocytes, albumin ^a^ and NUn score
Gao et al. [45]	Esophageal	Retrospective	2016–2017	100	96	12	38	Serum and drain	Amylase and prealbumin
Giulini et al. [46]	Esophageal	Retrospective	2015–2017	64	80	6	40	Serum and drain	Amylase and CRP
Gordon et al. [36]	Esophagogastric	Retrospective	2004–2014	0	145	13	NR	Serum	CRP
Hoeboer et al. [37]	Esophageal	Prospective	2011–2012	10	45	10	40	Serum	CRP, PCT and leucocytes ^a^
Ip et al. [38]	Esophageal	Retrospective	2012–2014	7	136	18	70	Serum	Lactate
Ji et al. [39]	Esophagogastric	Retrospective	2014	0	97	10	NR	Serum	CRP
Li et al. [40]	Esophageal	Retrospective	2013–2016	8	71	47	5	Serum and respiratory	CRP ^a^, PCT, leucocytes ^a^, albumin, Hb ^a^, PLT ^a^, BG, pO2 ^a^ and fiO2 ^a^
Machens et al. [48]	Esophageal	Retrospective	1992–1994	0	26	14	NR	Drain	amylase and ph ^a^
Miller et al. [55]	Esophageal	Retospective	2015–2016	100	45	3	27	Drain	Amylase
Noble et al. [41]	Esophageal	Retrospective	2005–2011	NR	258	26	156	Serum and score	CRP, leucocytes, albumin and NUn score
Paireder et al. [52]	Esophageal	Retrospective	2003–2014	28	258	32	177	Score	NUn score
Park et al. [42]	Esophageal	Retrospective	2009–2016	56	201	23	45	Serum	CRP and leucocytes ^a^
Perry et al. [49]	Esophageal	Retrospective	2007–2014	58	146	35	NR	Drain	Amylase
Plat et al. [54]	Esophageal	Prospective	2015–2016	NR	31	9	29	Urine	VOC
Schots et al. [50]	Gastric	Retrospective	2013–2017	70	107	8	71	Drain	Amylase and CRP ^a^
Song et al. [43]	Esophageal	Retrospective	2015–2016	67	183	16	50	Plasma	TNF-α, IL-2R, IL-6, IL-8 and IL-10
Yu et al. [51]	Esophageal	Retrospective	2014–2017	NR	99	10	17	Drain	Amylase

^a^ Statistical significance for detection AL not proven; ^b^ All studies include surgical resection for cancer and were mainly elective. AL, anastomotic leakage; NR, not reported, nCRT, neo-adjuvant chemoradiation therapy; MI, minimally invasive; CRP, C-reactive protein; PCT, procalcitonin; PN, per cent neutrophils; NUn score, 11.3894 + 0.005 (CRP) + 0186 (WCC) − 0.174 (Albumin); Hb, haemoglobin; PLT, platelet; BG, blood antigenemia; IL, interleukin.

**Table 2:** Reported definitions of anastomotic leakage.

Reference	Definition of Anastomotic Leakage
Asti et al. [32]	AL was suspected by the presence of clinical signs and confirmed by extravasation of oral contrast at gastrografin swallow study and/or computed tomography (CT), and/or visualization of anastomotic defect at upper gastrointestinal endoscopy.
Baker et al. [33]	AL was defined by contrast extravasation on postoperative CT esophagram or the presence of empyema on chest CT.
Berkelmans et al. [47]	AL was defined as any sign of leakage of the esophagogastric anastomosis on endoscopy, reoperation, and radiographic investigations, post mortal examination or when gastrointestinal contents were found in drain fluid.
Dutta et al. [34]	AL was confirmed by radiology (i.e., contrast enhanced multi-detector CT scan or conventional radiology with water soluble contrast), endoscopy, or during surgical exploration.
Edagawa et al. [44]	AL was defined as discontinuity of the esophagogastric anastomosis as detected by GIF, esophagography, or CT. The clinical significance of the leakage was not considered in this study.
Ellebaek et al. [53]	AL was suspected by the presence of clinical symptoms or alterations in blood samples indicating complications and checked by CT scan with oral contrast, followed by endoscopy.
Findlay et al. [35]	AL was defined in two ways: first as clinical or radiological evidence of a leak plus symptoms and second as any clinical or radiological evidence of a leak, irrespective of symptoms.
Gao et al. [45]	AL was defined as a gastroesophageal defect involving esophagus, anastomosis and conduit.
Giulini et al. [46]	AL was defined as a full-thickness lesion involving the anastomosis or the gastric conduit (staple line) requiring intervention or reiteration (grade III complication according to the Clavien–Dindo Classification) occurring within postoperative day (POD) 5.
Gordon et al. [36]	AL was defined as extravasation of oral contrast on cross-sectional imaging or an anastomotic defect visualized intraoperatively on return to theatre. Endoscopy was not used to diagnose AL.
Hoeboer et al. [37]	AL was defined as esophago-enteric leak confirmed by endoscopy or esophageal contrast videography that requires local treatment, surgical treatment, or removal of conduit.
Ip et al. [38]	AL was diagnosed by the presence of enteric content in the chest drain, endoscopic visualization of a defect in the esophagogastric anastomosis, or by extravasation of oral contrast at fluoroscopy or CT.
Ji et al. [39]	When postoperative AL was suspicious, methylene blue was administered orally. If the fluid from the abdominal drain was contaminated with blue dye, diagnosis of AL was confirmed.
Li et al. [40]	AL was defined as disruption of the esophagogastric anastomosis, the gastric staple line, or both identified by radiographic contrast examination, operative exploration, or both. Established by 3 methods: (1) oral methylene blue, (2) oral contrast computed tomography, and (3) endoscopy or operative exploration.
Machens et al. [48]	Cervical leaks confirmed on exploration of the neck wound were termed ‘major’, in contrast to those ‘minor’ leaks identified only in routine contrast studies.
Miller et al. [55]	No definition of AL reported. Anastomotic integrity was tested by a fluoroscopic water-soluble contrast agent or barium contrast agent, or both. Thoracic CT scans were performed if a leak was suspected or not clearly demonstrated on the swallow.
Noble et al. [41]	AL was defined as a leak sufficient to cause symptoms and confirmed by radiology (contrast-enhanced multi-detector CT scan with on-table contrast or water-soluble contrast studies), endoscopy or surgical exploration.
Paireder et al. [52]	No clear definition of AL given. Some ALs were diagnosed with routine contrast swallow. Article work based on the NUn score in Findlay and colleagues [35].
Park et al. [42]	AL was defined as the disruption of the anastomosis that leads to outflow of the intraluminal content, which is obvious leaks, as well as leaks without the presence of any clinical symptoms but with only occult leaks detected with esophagography followed by chest CT.
Perry et al. [49]	AL was defined as clinical or radiologic evidence of a full-thickness gastrointestinal defect involving the esophagus, anastomosis, staple line, or conduit.
Plat et al. [54]	AL was defined as a full thickness defect involving the esophageal anastomosis (the Esophagectomy Complications Consensus Group).
Schots et al. [50]	AL was defined as any sign of leakage of the gastrojejunostomy or esophagojejunostomy, entero-enterostomy, duodenal stump, or pancreas diagnosed by CT scan, endoscopy, or during reoperation.
Song et al. [43]	AL was diagnosed when one of the three following conditions was met: (1) chest radiography or computerized tomography obtained the presence of intra-thoracic collection of swallowing contrast agent adjacent to the anastomosis; (2) extravasation of gastrointestinal tract content through a wound or drainage tube; (3) direct observation of AL by postoperative gastroscopy examination; (4) intraoperative diagnosis.
Yu et al. [51]	AL was defined as clinical or radiological evidence of a full-thickness gastrointestinal defect involving the esophagus, anastomosis, staple line or conduit.

**Table 3:** Summary of very good diagnostic accuracy of CRP measurements.

Reference	Biomarker	POD	Cut-Off Values	AUROC	Sensitivity	SPECIFICITY	NPV	PPV	AL (n)
Asti et al. [32]	CRP	5	83 mg/L	0.818	89.3%	60.8%	97.7%	23.1%	29/243
Dutta et al. [34]	CRP	3	180 mg/L	0.808	82.0%	63.0%	NR	NR	17/136
		4	180 mg/L	0.857	71.0%	83.0%	NR	NR	17/136
Giulini et al. [46]	CRP	2	299 mg/L	0.902	100.0%	75.0%	NR	NR	4/80
Gordon et al. [36]	CRP	2	209 mg/L	0.819	100.0%	64.0%	100.0%	21.0%	13/145
		3	190 mg/L	0.836	100.0%	61.0%	100.0%	20.0%	13/145
		6	154 mg/L	0.907	100.0%	80.0%	100.0%	33.0%	13/145
Hoeboer et al. [37]	CRP	Δ0-3	55	0.820	80.0%	80.0%	94.0%	50.0%	10/45
Ji et al. [39]	CRP	1	117 mg/L	0.994	90.0%	89.0%	NR	NR	10/97
		2	177 mg/L	0.908	90.0%	95.0%	NR	NR	10/97
		3	153 mg/L	0.936	90.0%	89.0%	NR	NR	10/97
		4	89 mg/L	0.917	90.0%	95.0%	NR	NR	10/97
		5	92 mg/L	0.881	90.0%	95.0%	NR	NR	10/97
Park et al. [42]	CRP (non-NT)	3	171.2 mg/L	0.822	69.2%	78.1%	NR	NR	15/156
	CRP (non-NT/MIE)	3	128.6 mg/L	0.800	83.3%	64.9%	NR	NR	NR/89
	CRP (non-NT/OE)	3	179.4 mg/L	0.834	71.4%	72.0%	NR	NR	NR/67

^a^ Fractional change, value day 3 divided by day 0; POD, postoperative day; AUROC, area under receiver operating curve; NPV, negative predictive value; PPV, positive predictive value; AL (n), patients with AL/study population; CRP, C-reactive protein; Non-NT, non-neoadjuvant therapy; MIE, minimally invasive esophagectomy; OE, open esophagectomy; NR, not reported.

**Table 4:** Summary of other (very) good biomarkers (AUROCs > 0.700).

Reference	Biomarker	POD	Cut-off Values	AUROC	Sensitivity	Specificity	NPV	PPV	AL (n)
Gao et al. [45]	Prealbumin	5	128 g/L	0.824	100.0%	50.0%	NR	NR	12/96
Hoeboer et al. [37]	PCT	3	0.35 ng/ml	0.860	67.0%	80.0%	87.0%	55.0%	10/45
Noble et al. [41]	NUn score	4	10	0.801	95.0%	49.0%	NR	NR	26/258
Asti et al. [32]	PCT	5	0.380 ng/ml	0.751	77.8%	71.4%	94.2%	35.0%	29/243
Hoeboer et al. [37]	PCT	1	1.82 ng/ml	0.760	22.0%	100.0%	83.0%	100.0%	10/45
Li et al. [40]	BG	Any	93 pg/mL	0.773	61.7%	83.3%	72.3%	66.7%	47/71
	PCT	Any	3 ng/mL	0.752	72.3%	67.7%	63.8%	83.3%	47/71
	PCT × BG	Any	261	0.773	72.3%	91.7%	72.3%	91.7%	47/71
Noble et al. [41]	Albumin	5	22.5 g/L	0.742	76.0%	56.0%	NR	NR	26/258
	WCC	5	8.95 (x10/L)	0.715	78.0%	58.0%	NR	NR	26/258
	NUn score	5	10	0.796	88.0%	55.0%	NR	NR	26/258
Song et al. [43]	IL-6	1	74.6 pg/mL	0.735	100.0%	45.7%	NR	NR	16/183
	IL-8	1	61.1 pg/mL	0.720	60.0%	45.7%	NR	NR	16/183
	IL-10	1	17.2 pg/mL	0.784	66.7%	84.8%	NR	NR	16/183
Giulini et al. [46]	Amylase	1	335 IU/L	0.814	75.0%	100.0%	NR	NR	4/80
Schots et al. [50]	Amylase	1	750 IU/L	0.703	71.4%	81.4%	96.0%	31.3%	8/293
		Optimal ROC	1000 IU/L	0.805	71.4%	94.9%	96.6%	62.5%	8/293
Yu et al. [51]	Amylase	3	544 IU/L	0.778	66.7%	83.8%	NR	NR	10/99

POD, postoperative day; AUROC, area under receiver operating curve; NPV, negative predictive value; PPV, positive predictive value; AL (n), patients with AL/study population; Optimal ROC, optimal receiver operating curve with random cut-off value given the highest AUROC; PCT, procalcitonin; BG, blood G; WCC, white cell count; IL, Interleukin.
